# IASM: A System for the Intelligent Active Surveillance of Malaria

**DOI:** 10.1155/2016/2080937

**Published:** 2016-07-31

**Authors:** Xinlei Wang, Bo Yang, Jing Huang, Hechang Chen, Xiao Gu, Yuan Bai, Zhanwei Du

**Affiliations:** ^1^School of Computer Science and Technology, Jilin University, Changchun 130012, China; ^2^Key Laboratory of Symbolic Computation and Knowledge Engineering of Ministry of Education, Changchun 130012, China

## Abstract

Malaria, a life-threatening infectious disease, spreads rapidly via parasites. Malaria prevention is more effective and efficient than treatment. However, the existing surveillance systems used to prevent malaria are inadequate, especially in areas with limited or no access to medical resources. In this paper, in order to monitor the spreading of malaria, we develop an intelligent surveillance system based on our existing algorithms. First, a visualization function and active surveillance were implemented in order to predict and categorize areas at high risk of infection. Next, socioeconomic and climatological characteristics were applied to the proposed prediction model. Then, the redundancy of the socioeconomic attribute values was reduced using the stepwise regression method to improve the accuracy of the proposed prediction model. The experimental results indicated that the proposed IASM predicted malaria outbreaks more close to the real data and with fewer variables than other models. Furthermore, the proposed model effectively identified areas at high risk of infection.

## 1. Introduction

*(A) Background*. Malaria, a life-threatening infectious disease, usually spreads to humans via infected mosquitos. Malaria has been extensively researched. In fact, five research projects concerning malaria have won the Nobel Prize. Recently, in 2015, the Chinese pharmacist Youyou Tu won the Nobel Prize in physiology and medicine after discovering that the extract artemisinin used in traditional Chinese medicine (TCM) can effectively inhibit the malaria parasite. Articles concerning malaria are frequently published in top research journals including* Nature and Science* [1–8]. For example, Gardner et al. [1] suggested that biological methods should be used to prevent and treat malaria; Walker et al. analyzed the malignant impact of malaria using real data [9, 10]. The current malaria studies have consistently demonstrated that prevention is more effective and efficient than treatment. 

*(B) Related Work*. Numerous methods of malaria prevention and prediction have been developed [11–20]. In addition, many studies concerning the infectious process of malaria have been conducted. However, further insight regarding the spread of malaria could be obtained through the development of prediction models. For example, Yang et al. developed the prediction model NetEpi (Network Epidemic) in order to identify the methods of malaria transmission and predict the spread of infection [12]. In addition, a spatial transmission model representing both the heterogeneous transmission potential of* P. vivax* at individual locations and the mobility of infected populations among different locations was developed using neural networks in order to identify transmission networks based on surveillance data [16]. Gomez-Elipe et al. use ARIMA (autoregressive integrated moving average) to predict the malaria infections with time series of monthly notifications of malaria cases from local health facilities, data from rain and temperature records, and the normalized difference vegetation index (NDVI) [19]. Our research team has done some research on malaria prediction [11, 17]. However, all of these researches focus on models and algorithms to study malaria prediction. 

*(C) Motivation*. Effective prevention and control measures are needed to reduce the number of malaria cases as well as monitor current and potential outbreaks. Thus, a visualization system for the fast, efficient, and real-time detection of malaria is necessary. Knoema, an online system, provides a query of WHO World Malaria Statistics data ranging from 1990 to 2014 [21]. In GeoSurveillance, a spatial statistical method and basic geographic function are used to review and assess the risk of spatial clustering and set monitoring [22]. However, these surveillance systems only predicted approximately 14% of worldwide malaria cases in 2012 and exhibit poor prediction accuracy when there is insufficient data [23, 24]. In addition, these systems obtain information passively from hospital data. Passive surveillance is both time-consuming and costly since it entails the collection of individual surveys from all affected and potentially affected regions. Therefore, an active surveillance model that requires limited information is needed to achieve optimal malaria prevention and control. 

*(D) Contributions*. The following contributions are presented in this paper.

Due to the abovementioned problems of the prior systems, based on the work of existing laboratories, we have completed the visualization system that can be used for active surveillance. The existing data shows that our system is the first to achieve proactive monitoring visualization system:

(i) Due to the abovementioned problems of the prior systems, based on the existing work of our team, an active surveillance system was developed. In the past, medical institutions have acquired information passively by collecting data from public health agencies and patients, inhibiting the accurate and timely detection of high-risk areas. In this study, an active malaria system was developed by combining data, prediction results, and top-*k* algorithm. In the proposed active surveillance system, individual incidences of infection were identified via active searching and surveys. And existing researches show that our system is the first visualization system with active surveillance. For example, through active surveillance, seven towns in Tengchong County that comprised 70% of the malaria cases in that county were identified. Then, medical resources were distributed to those seven towns in order to control the spread of malaria to other areas. Details regarding this process can be found in Section 3.3.
(ii) According to the data analysis, as many as 98% of the malaria cases in Tengchong, China, were imported from Myanmar. Thus, the number of workers from Myanmar, the probability of people from Tengchong working in Myanmar becoming infected, and the number of people returning from Myanmar influenced the prediction results. In order to account for this information, a logistic regression model, an improved population radiation model [25], a VCAP model [26], and climatological factors were introduced to the proposed system.
(iii) Based on our researches of active surveillance, NAS algorithm is proposed. The dimensionality of the input data was reduced in order to achieve good prediction results with less data, thereby improving the accuracy of the proposed system. Furthermore, the stepwise regression method was added to the prediction model in order to obtain prediction results using only five attributes. Details regarding this process can be found in Section 3.3.

The rest of the paper is structured as follows. In Section 2, the framework of intelligent surveillance is presented. Then, the design of the back-end system and the process used to reduce the redundancy of the socioeconomic attribute values in the prediction model are described in Section 3.

## 2. IASM Design

Our research group has been working on forecasting infectious disease and has proposed the concept of active surveillance for reasonable allocation when medicine and human resources are limited. Our previous work about active surveillance planning has been published in AAAI conference in 2014 [11].

**Figure 1:**
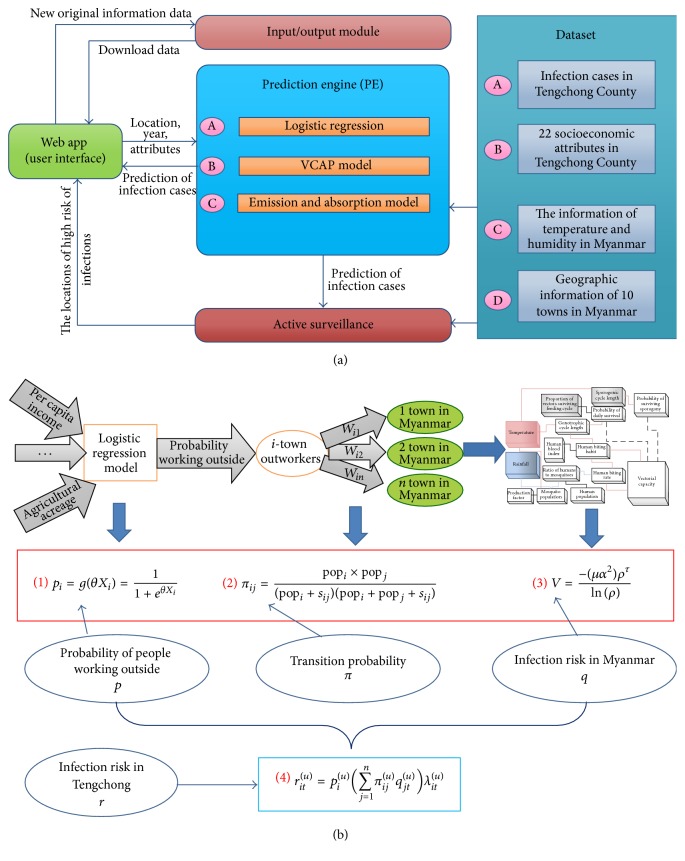
(a) The overall architecture of the IASM. Users can select a location, year, and socioeconomic and environmental attributes using a Web UI. The information submitted by the user is then sent to the geographic information display and prediction engine models. The first model displays the location information on a map. Next, the prediction engine generates the prediction results using the input information and datasets. Then, the areas at high risk of infection are identified based on the prediction results via active surveillance. (b) Detailed structure of a PE module with a VCAP extension and some equations [11].

The system proposed in this study is displayed in Figure 1. The proposed system consists of a user interface (UI), geographic information display, prediction engine (PE), and active surveillance model. The geographic information display, prediction engine (PE), and active surveillance model are all function modules. As shown in Figure 1(a), a user can interact with the function modules by selecting a location, time, and various attributes using the user interface (UI). The corresponding results are then generated by the selected modules based on existing data and algorithms. Then, the active surveillance model displays the selected function as well as the prediction results obtained by the prediction engine (PE) model. Details regarding the prediction engine (PE) are displayed in Figure 1(b). Since most existing software includes a user interface (UI) and geographic information display, details regarding these models will not be discussed in this paper. However, details regarding the prediction engine (PE) and active surveillance model are provided herein. The attributes are also optimized in this paper.

### 2.1. Prediction Engine (PE)

The PE module consisted of a logistic regression model, improved population radiation model, and active surveillance model. These three models are discussed in the following passages.

#### 2.1.1. Logistic Regression Model

A logistic regression model is constructed to describe the relationships among the socioeconomic attributes of a selected location as well as the probability that the people living at that location will leave the area for work [11]. In formula (1) shown in Figure 1(b), for a location *x*_*i*_, *X*_*i*_ = (*x*_*i*1_,…, *x*_*in*_) and *x*_*ij*_ denotes the value of attribute *α*_*i*_ for location *x*_*i*_. In addition, *θ* = (*θ*_1_,…, *θ*_*n*_) denotes the weight of each attribute, and *p*_*i*_ denotes the probability that the people living in the selected region will leave that region for work. In this study, 22 socioeconomic attributes that could influence whether the people of a region leave that region for work are considered.

#### 2.1.2. Improved Population Radiation Model

The probability that the people working outside work in a certain region can be estimated as [11, 25]. In formula (2) shown in Figure 1(b), pop_*i*_ and pop_*j*_ denote the populations of the source location *x*_*i*_ and target location *y*_*i*_, respectively. In addition, *s*_*ij*_ denotes the total population within a certain radius *r*_*ij*_ (the distance between *x*_*i*_ and the target location) of the selected location.

#### 2.1.3. VCAP

The risk of infection with malaria can be estimated based on the humidity and temperature of the selected location as [26]. In formula (3) shown in Figure 1(b), *V* represents the vector capacity of the selected area, *μ* denotes the equilibrium mosquito density per human, *α* denotes the expected number of bites on the people in that region per mosquito per day, *ρ* denotes the probability of a mosquito surviving an entire day, and *τ* denotes the extrinsic incubation period of malaria parasites or the time required to complete the extrinsic cycle. All the above parameters of the VCAP could be influenced by temperature and rainfall [27].

Furthermore, the risk of infection of location *y* can be estimated as [28]

(1) $$\begin{array}{c} q=\frac{\beta V-\sigma }{\beta V+\sigma \left(\alpha /\eta \right)}, \end{array}$$

where *β* denotes the probability that an uninfected human will become infected after being bitten by an infectious mosquito, *σ* denotes the recovery rate of humans, and *η* denotes the per capita daily death rate of a mosquito, which is equal to ln⁡(*ρ*).

Based on the above analysis, the risk of infection of a source location *z*_*i*_ after a time interval *t* in a certain year *u* can be expressed as formula (4) shown in Figure 1(b).

The total surveillance data of year *Y* can be represented as a cube tensor denoted by *C* = [*c*_*uit*_]_*Y*×*M*×*T*_, where *c*_*uit*_ denotes the number of incidences reported at location *x*_*i*_. Here, *θ* is a dynamic variable with a value of *θ*_*it*_^*u*^ that varies at different time.

For this study, we assume that the observed variable is not the same as the truth and that it has a Gaussian distribution centered at the observation [29]. *θ*_*it*_^*u*^ between two continuous times would not change too much. Thus, particle filter method is used here, which is able to meet the two above requests.

The observation error variance (OEV) of week *k*, or OEV_*k*_, can be defined as

(2) $$\begin{array}{c} {\text{OEV}}_{k}=1\times 10^{1}+\frac{{\left(\sum_{j=k-3}^{k-1}\left({\text{obs}}_{j}/3\right)\right)}^{2}}{5}, \end{array}$$

where obs_*j*_ is the observation of week *j*. The above equation indicates that the value of OEV_*k*_ is proportional to the average observation of the 3 preceding seasons. This Gaussian distribution, which is primarily based on the algorithm in [30], was used during the particle filter process.

### 2.2. Active Surveillance

The number of infected cases in each area can be determined based on the prediction results of that area. In addition, the areas at high risk of infection can be identified by ranking the prediction results of the regions. This process is especially significant in that when resources are limited, areas at high risk of infection can be treated with the top-*k* methods in order to more effectively prevent and control malaria outbreaks.

## 3. Framework 

Epidemiological research has a long history. If the network structure of the spread model of an epidemic is known, a supplemented propagation model and prediction information can be obtained, allowing for early warnings.

However, in reality, directly identifying the spread network structure of an epidemic is difficult since the infectors and those at risk of becoming infected are not always clearly defined. However, the spatiotemporal-series data of an epidemic can be directly observed. Thus, hidden trends in this data could be used to identify the spread network structure of an epidemic. In order to visualize these prediction results, an IASM system is proposed.

IASMs provide frameworks for the intelligent surveillance of input and output module, the prediction results of disease, and active surveillance. Active surveillance functions can be obtained using prediction results. We create the program as follows:

 main(){
   IASM.io_data();
   //user can input new data and download data.
   IASM.malaria_prediction();
   // user can predict malaria outbreak trend
   IASM.malaria_active_surveillance();
   //user can selected some key areas to set sentinel by active surveillance
 }

### 3.1. Input/Output Module

**Figure 2:**
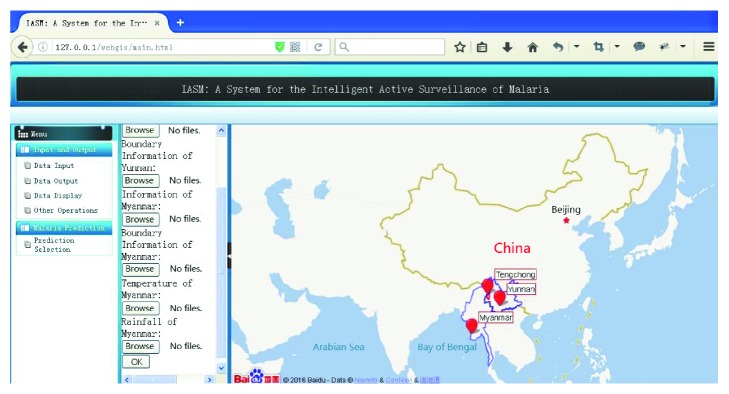
Data input/output module interface.

As shown in Figure 2, the user interface of IASM is divided into two areas. The left side is designed as a function interface, which is primarily for selecting the display or controlling the background calculation command. Background operation result set can be transformed to graphical interface in the right side of the user interface. A more intuitive understanding and analysis can be obtained.

In function interface (Menu), there is an “input and output” module. We would like to introduce four options (data input, data output, display, and other operations) of this module.

#### 3.1.1. Data Input

User can input new original information data to the server by user interface. Through the HTTP protocol, the front page transfers original information data to the background PHP scripts. Then, background PHP scripts analyze and deal with the data. And according to the predefined formats, the results would be stored in database. See Algorithm 1.

After the data input, the user can select operation parameters of the model to perform the operation, such as learning years and prediction years. Through this interface, iterative operation can be done with existing database. The result of the operation would be saved to the database.

#### 3.1.2. Data Output

In the simulation process, different result sets of data can be produced. These results would be stored to the database and given a unique number. Depending on the different numbers, the corresponding result set can be downloaded from the server for detailed analysis. See Algorithm 2.

In order to enable users to get an intuitive understanding, the relevant data would be displayed on the right area with charts.

#### 3.1.3. Display

**Figure 3:**
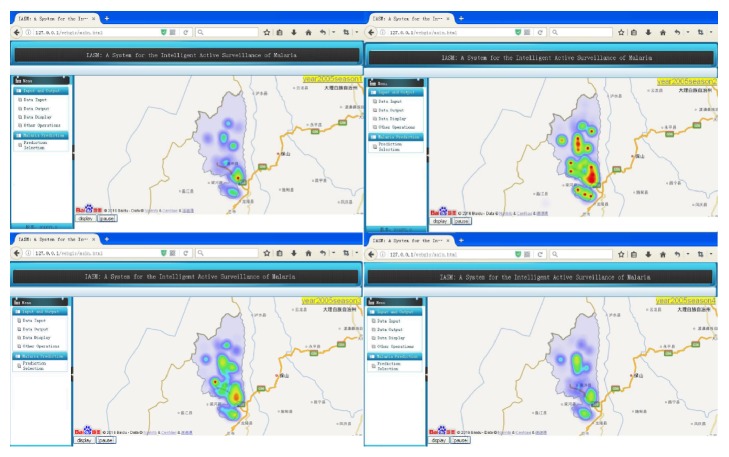
Screenshots of the dynamic outbreak interface of malaria.

For the season of time granularity, it will display the number of infected cases in the right area with heat map. By the way of image, the relationship with time, space, and infection situation would be displayed visually. One can zoom in or out (such as city, county, and village) on the infection map to observe different levels of administrative regions, as shown in Figure 3.

#### 3.1.4. Other Operations

**Figure 4:**
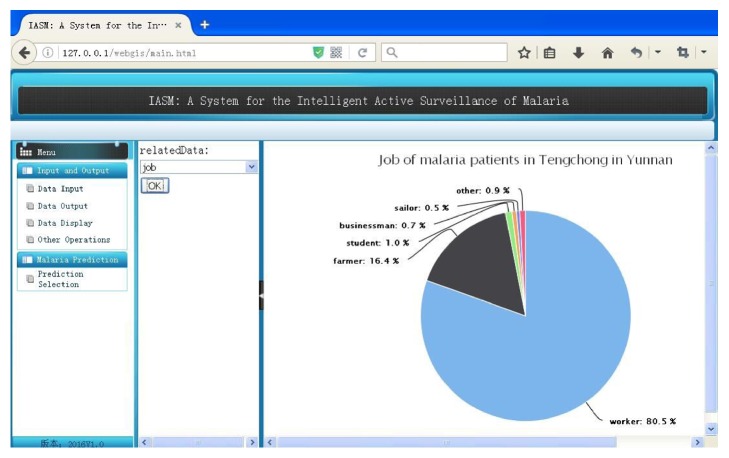
Screenshot of crowd classification interface with related data analysis.

This module displays the original information data in the operation process with different charts, such as pie charts and histograms. Through different charts, the user could get some hidden data relationships. Part of the interface is shown in Figure 4.

### 3.2. Disease Prediction Results

**Figure 5:**
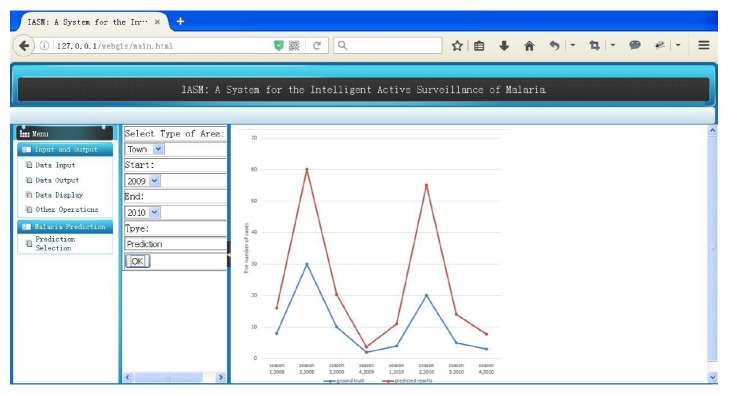
Screenshot of the infection prediction results for Qu Stone Town from 2009 to 2010. The predicted results are depicted by the red line, while the ground true data is depicted by the blue line. Both sets of data can be downloaded.

Research concerning infectious diseases involves complex biological information and environmental factors, such as temperature and humidity, which can influence the incidence rate. However, both environmental and socioeconomic factors can be used to predict whether an area is at risk of infection. Socioeconomic factors have been largely neglected in previous studies. In contrast, 22 socioeconomic factors, such as the reasons why people work outside, were considered in the system developed in this study. Since different combinations of socioeconomic and environmental attributes could yield varying prediction results, the influence of the various attributes on the prediction results was included in the proposed system, as shown in Figure 5. As shown in this figure, in function interface (Menu), there is a “Malaria Prediction” module, which has only one option (Prediction Selection). A user can construct a prediction model by selecting the type of area, the time range, and the prediction type.

### 3.3. The Strategies of Active Surveillance for Controlling Malaria Outbreak

**Figure 6:**
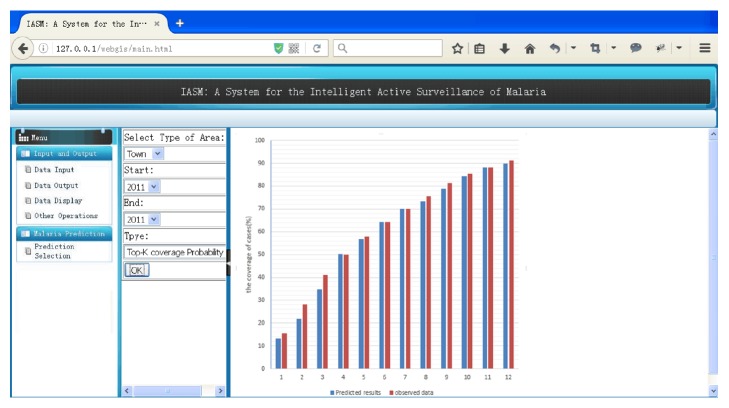
Coverage rate prediction for top-k towns from *k* = 1 to *k* = 12. This screenshot displays the coverage of cases results of ranking 1 to 12 towns in Tengchong County in 2011. The blue histograms are estimated with the predicted results by active surveillance with the coverage probability and the principle of top-*k*. The red histograms are drawn with the ground truth data.

Active surveillance can be used to identify areas at high risk of infection. For example, certain resources, such as time, money, and medical equipment, are limited, and medical workers can use active surveillance to monitor the spread of malaria, as shown in Figure 6. In “Malaria Prediction” module, active surveillance can be implemented by selecting the type of area, the time range, and the type of “top-*k* coverage probability.”

## 4. An Empirical Study in Tengchong County with NAS

Surveillance data concerning the monthly number of malaria cases in Tengchong County over four years (2007–2010) was obtained from annual reports provided by the National Institute of Parasitic Disease and the Chinese CDC. A total of 221 villages were included in the data. The annual demographic data was obtained from the Chinese Natural Resources Database. The socioeconomic data, including a total of 22 socioeconomic factors, was obtained from annual reports issued by the Tengchong government. Using these data, 18 towns were selected as the source locations for the purposes of this study.

Since official data is not accessible in Myanmar, obtaining data was difficult. Most of the selected target locations were cities or towns located near the Yunnan-Myanmar international border [11]. The temperature and rainfall data of these locations were obtained via three sources, that is, the IRI/LDEO Climate Data Library, TRMM (Tropical Rainfall Measuring Mission), and MODIS (MODerate-resolution Imaging Spectroradiometer). The remaining two datasets were provided by NASA. The useful data was extracted using the remote sense image processing software ENVI (ENvironment for Visualizing Images). The geographical and transportation data were obtained from Google Earth.

Surveillance data obtained from 2007 to 2009 was used for learning and data obtained during 2010 was used for testing. Specifically, the socioeconomic factors influencing the number of imported incidences were identified, and the accuracy of the prediction system and effectiveness of active surveillance under different coverage thresholds were investigated.

**Figure 7:**
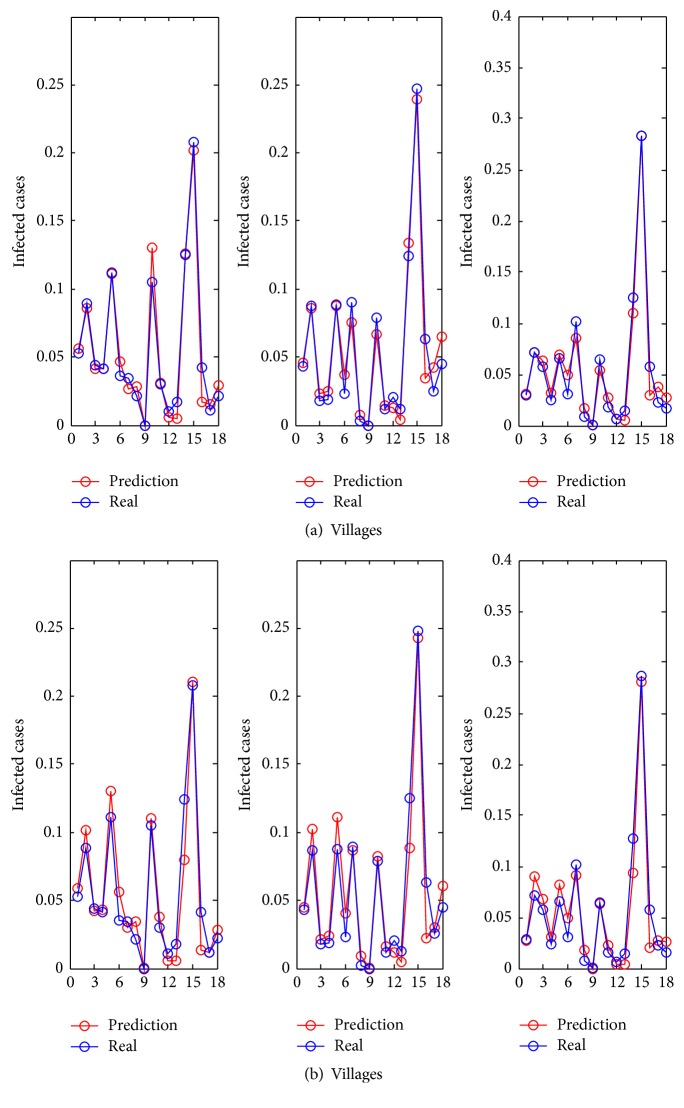
Distributions of the infected cases of 18 towns in 2010. The fitted results are depicted by the red line, while the ground truth data is depicted by the blue line. (a) The infected cases' distribution with 22 attributes. (b) The infected cases' distribution with 5 attributes.

Using the estimated clustering indicator, the 18 towns in Tengchong were clustered into 6 groups. Although 22 socioeconomic attributes of Tengchong County were available, not all of these factors were needed in the regression model. Using the stepwise regression method, 5 attributes, that is, the village population, total meat output, natural population growth rate, rural employed population, and current output, were selected. In order to demonstrate that the 5 selected attributes could be used to achieve prediction results similar to those of our previous study [11], the proposed method was applied to the data obtained in 2010 using those attributes. In Figure 7, the blue line represents the actual data, while the red line represents the results predicted using the proposed method with 22 and 5 attributes, respectively.

**Figure 8:**
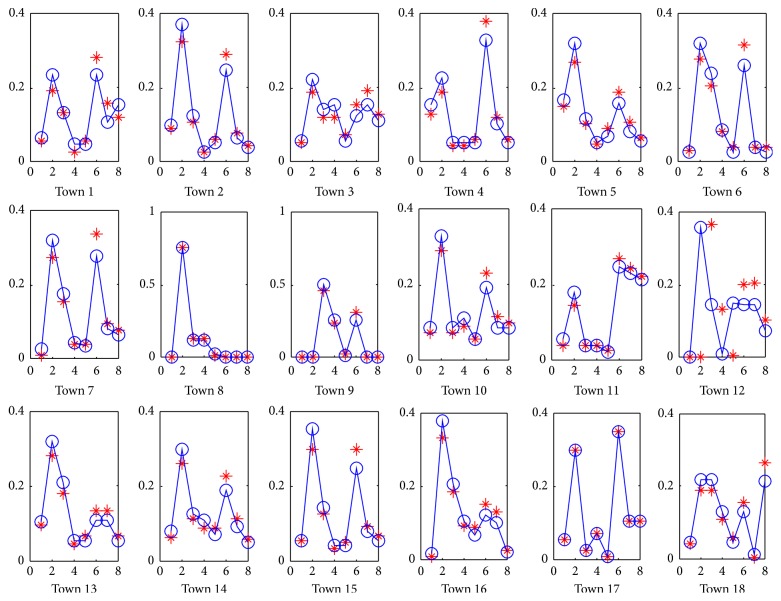
Prediction results of 2010 by using the data of years 2007–2009.

The prediction results are shown in Figure 8. In this figure, the red point represents the actual data, and the blue line represents the predicted values using the proposed method with 5 attributes. The *x*-axis denotes 8 seasons ranging from 2009 to 2010, and *y*-axis denotes the infection risk which is normalized as *C*_*it*_/∑_*i*_ *C*_*it*_, where *C*_*it*_ is the infected cases of location *i* at time *t*. The first four seasons denote the fitting results, while the last four seasons denote the results predicted using the parameters inferred from the 2009 data. As we see, the prediction fits the ground truth very well even for the locations with insufficient surveillance data. This implies that the proposed method is suitable to make a prediction in terms of infection risks.

**Figure 9:**
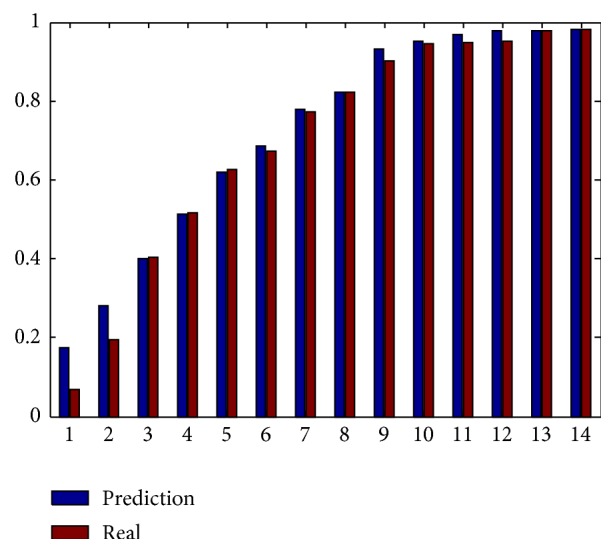
Comparison of the top-*k* towns selected using the proposed method (left) and the benchmark top-*k* towns (right).

The top-*k* towns selected using the proposed method were also compared to the benchmark top-*k* towns based on their coverage rates, as shown in Figure 9. In the proposed method, the four most important towns are selected. Then, the remaining 14 towns are ranked. In Figure 9, the *x*-axis represents the top-*k* towns of the remaining 14 towns. As shown by this figure, the proposed method yielded coverage rates similar to those of the benchmark top-*k* towns from top 3 to top 14.

**Figure 10:**
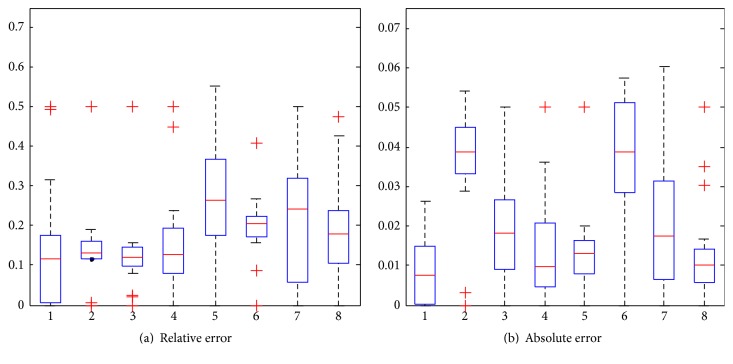
Boxplot of prediction errors. (a) RE of infection risks prediction. (b) AE of infection risks prediction.

To test the stability of the proposed method when estimations vary, we plot the confidence intervals of prediction errors in terms of RE (relative error) and AE (absolute error). Hence, ${RE}_{i}=\left|y_{i}-{\hat{y}}_{i}\right|/y_{i}$ and ${AE}_{i}=\left|y_{i}-{\hat{y}}_{i}\right|$, where *y*_*i*_ and ${\hat{y}}_{i}$ represent the ground truth and the prediction of infected cases of location *i* and |·| denotes the absolute value of a scalar. As shown in Figure 10, the *x*-axis indicates eight seasons of 2009 and 2010, and *y*-axis is the prediction error. Specifically, the bottom and top of the boxes correspond to the 25th and 75th percentiles, and the horizontal segment, that is, the red line, indicates the median. The ends of the whiskers correspond to the 5th and 95th percentiles. The red markers are outliers located outside the 90% confidence interval, that is, events falling below the 5th percentile or above the 95th percentile. As we see, for all the predicted seasons, the ranges of confidence intervals in terms of relative errors are less than 25%, as shown in Figure 10(a). Specifically, for the seasons of 1, 4, 5, and 8, the ranges are less than 20%, and for 2, 3, and 6 the ranges are less than 5%. This indicates that the proposed method is stable to make a prediction in most of the cases. In addition, the mean errors for all seasons are less than 25%, suggesting that the proposed method has the ability to make a stable prediction for all seasons accurately. Similarly, Figure 10(b) shows the confidence intervals of prediction errors for eight seasons.

## 5. Conclusions

In this paper, a malaria surveillance system was developed in order to monitor and predict the transmission of malaria in Tengchong County of Yunnan Province, China. Active surveillance was used to identify areas at high risk of infection based on socioeconomic attributes using a logistic regression model. The proposed system compensated for a lack of data. In addition, the particle filter method was used to estimate the values of the parameters based on the differences in the observation error variance values of two instances and the dynamic change between two continuous times.

The system was then applied to data collected from 18 towns in Tengchong County. The experimental results indicated that the proposed system yielded prediction results similar to the real data. Moreover, the redundancy of the socioeconomic attribute values of the prediction model was reduced by greater than 50%, while maintaining a similar prediction accuracy. Therefore, the proposed system could be used to effectively monitor and control malaria outbreaks in Tengchong County.

In future studies, a new method capable of utilizing hidden information to effectively predict and monitor malaria cases will be developed.

## Figures and Tables

**Algorithm 1:**
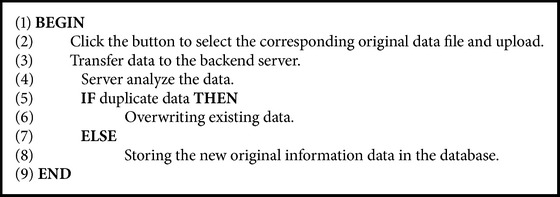
〈Data input〉.

**Algorithm 2:**
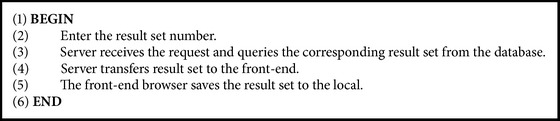
〈Data output〉.

## References

[1] Gardner M. J., Hall N., Fung E. (2002). Genome sequence of the human malaria parasite *Plasmodium falciparum*. *Nature*.

[2] Aebersold R., Mann M. (2003). Mass spectrometry-based proteomics. *Nature*.

[3] Paddon C. J., Westfall P. J., Pitera D. J. (2013). High-level semi-synthetic production of the potent antimalarial artemisinin. *Nature*.

[4] Ariey F., Witkowski B., Amaratunga C. (2014). A molecular marker of artemisinin-resistant *Plasmodium falciparum* malaria. *Nature*.

[5] Dondorp A. M., Nosten F., Yi P. (2009). Artemisinin resistance in *Plasmodium falciparum* malaria. *The New England Journal of Medicine*.

[6] Ashley E. A., Dhorda M., Fairhurst R. M. (2014). Spread of artemisinin resistance in *Plasmodium falciparum* malaria. *The New England Journal of Medicine*.

[7] Trager W., Jensen J. B. (1976). Human malaria parasites in continuous culture. *Science*.

[8] Hoffmann J. A., Kafatos F. C., Janeway C. A., Ezekowitz R. A. B. (1999). Phylogenetic perspectives in innate immunity. *Science*.

[9] Walker S. P., Wachs T. D., Meeks Gardner J. (2007). Child development: risk factors for adverse outcomes in developing countries. *The Lancet*.

[10] Lozano R., Naghavi M., Foreman K., Lim S., Shibuya K., Aboyans V. (2013). Global and regional mortality from 235 causes of death for 20 age groups in 1990 and 2010: a systematic analysis for the Global Burden of Disease study 2010. *The Lancet*.

[11] Yang B., Guo H., Yang Y., Shi B., Zhou X., Liu J. Modeling and mining spatiotemporal patterns of Infection risk from heterogeneous data for active surveillance planning.

[12] Yang X., Liu J., Zhou X. N., Cheung W. K. (2014). Inferring disease transmission networks at a metapopulation level. *Health Information Science and Systems*.

[13] Liu J., Yang B., Cheung W. K., Yang G. (2012). Malaria transmission modelling: a network perspective. *Infectious Diseases of Poverty*.

[14] Shi B., Liu J., Zhou X.-N., Yang G.-J. (2014). Inferring *Plasmodium vivax* transmission networks from tempo-spatial surveillance data. *PLoS Neglected Tropical Diseases*.

[15] Xia S., Liu J., Cheung W. (2013). Identifying the relative priorities of subpopulations for containing infectious disease spread. *PLoS ONE*.

[16] Shi B., Tan Q., Zhou X.-N., Liu J. (2015). Mining geographic variations of *Plasmodium vivax* for active surveillance: a case study in China. *Malaria Journal*.

[17] Gu X., Chen H., Yang B. Heterogeneous data mining for planning active surveillance of malaria.

[18] Loha E., Lindtjørn B. (2010). Model variations in predicting incidence of *Plasmodium falciparum* malaria using 1998–2007 morbidity and meteorological data from south Ethiopia. *Malaria Journal*.

[19] Gomez-Elipe A., Otero A., Van Herp M., Aguirre-Jaime A. (2007). Forecasting malaria incidence based on monthly case reports and environmental factors in Karuzi, Burundi, 1997–2003. *Malaria Journal*.

[20] Hanf M., Adenis A., Nacher M., Carme B. (2011). The role of El Nĩo southern oscillation (ENSO) on variations of monthly Plasmodium falciparum malaria cases at the cayenne general hospital, 1996-2009, French Guiana. *Malaria Journal*.

[21] http://cn.knoema.com/WHOWMS2014/who-world-malaria-statistics-2014.

[22] Yamada I., Rogerson P. A., Lee G. (2009). GeoSurveillance: a GIS-based system for the detection and monitoring of spatial clusters. *Journal of Geographical Systems*.

[23] http://www.who.int/mediacentre/factsheets/fs094/en/.

[24] http://www.who.int/mediacentre/news/releases/2012/malaria_20121217/en/.

[25] Simini F., González M. C., Maritan A., Barabási A.-L. (2012). A universal model for mobility and migration patterns. *Nature*.

[26] Ceccato P., Vancutsem C., Klaver R., Rowland J., Connor S. J. (2012). A vectorial capacity product to monitor changing malaria transmission potential in epidemic regions of Africa. *Journal of Tropical Medicine*.

[27] Paaijmans K. P., Read A. F., Thomas M. B. (2009). Understanding the link between malaria risk and climate. *Proceedings of the National Academy of Sciences of the United States of America*.

[28] Smith D. L., McKenzie F. E. (2004). Statics and dynamics of malaria infection in Anopheles mosquitoes. *Malaria Journal*.

[29] Yang W., Karspeck A., Shaman J. (2014). Comparison of filtering methods for the modeling and retrospective forecasting of influenza epidemics. *PLoS Computational Biology*.

[30] Arulampalam M. S., Maskell S., Gordon N., Clapp T. (2002). A tutorial on particle filters for online nonlinear/non-Gaussian Bayesian tracking. *IEEE Transactions on Signal Processing*.

